# Phytotoxicity of Bisphenol A to *Allium cepa* Root Cells Is Mediated through Growth Hormone Gibberellic Acid and Reactive Oxygen Species

**DOI:** 10.3390/molecules28052046

**Published:** 2023-02-22

**Authors:** Valerija Vujčić Bok, Marko Gerić, Goran Gajski, Sanja Gagić, Ana-Marija Domijan

**Affiliations:** 1 Department of Pharmaceutical Botany, Faculty of Pharmacy and Biochemistry, University of Zagreb, Kovačićeva 1, 10000 Zagreb, Croatia; 2 Mutagenesis Unit, Institute form Medical Research and Occupational Health, Ksaverska c. 2, 10000 Zagreb, Croatia

**Keywords:** bisphenols, root growth, plant hormones, genotoxicity, malondialdehyde, protein carbonyls, polyphenols

## Abstract

The aim of this study was to test the phytotoxicity and mode of action of bisphenol A (BPA) on *Allium cepa* using a multibiomarker approach. *A. cepa* roots were exposed to BPA in concentration range 0–50 mg L^−1^ for 3 days. BPA even in the lowest applied concentration (1 mg L^−1^) reduced root length, root fresh weight, and mitotic index. Additionally, the lowest BPA concentration (1 mg L^−1^) decreased the level of gibberellic acid (GA_3_) in root cells. BPA at concentration 5 mg L^−1^ increased production of reactive oxygen species (ROS) that was followed by increase in oxidative damage to cells’ lipids and proteins and activity of enzyme superoxide dismutase. BPA in higher concentrations (25 and 50 mg L^−1^) induced genome damage detected as an increase in micronucleus (MNs) and nuclear buds (NBUDs). BPA at >25 mg L^−1^ induced synthesis of phytochemicals. Results of this study using multibiomarker approach indicate that BPA is phytotoxic to *A. cepa* roots and has shown genotoxic potential to plants, thus its presence in the environment should be monitored.

## 1. Introduction

Bisphenol A (BPA; 4,4′-(propane-2,2-diyl)diphenol) is a carbon-based synthetic chemical that is used as the intermediary in the production of plastic-based materials such as plastic bottles including baby bottles, food cans, electronic items, and medical equipment [1,2,3]. Due to the high demand for plastic-based materials in everyday life, BPA enters the environment, where it becomes ubiquitous. It is estimated that 1 million pounds of BPA are released annually into the environment, mainly due to the polymer industry [4,5]. Consequently, BPA is detected in the atmosphere, in rivers, seawater, and in soil [6,7,8,9]. It is demonstrated that BPA can reside in soil for some time [10] and from the soil, plants easily absorb BPA [11,12]. In 2010, the European Union released a risk assessment report on BPA in the terrestrial ecosystem and indicated that further investigations on the impact of BPA on plants are needed [10].

Studies so far have reported that BPA inhibits the growth or germination of several plants such as *Glycine max* (soyabean) [12], *Pisum sativum* (pea) [13], *Oryza sativa* (rice) [5], *Cicer arietinum* (chickpea) [14], *Nicotiana tabacum* (tobacco) [15], *Vicia faba* (faba bean) [16], and *Allium cepa* (onion) [17]. It is found that BPA interferes with the production of plants’ hormones [12], affects photosynthesis by inhibiting carbon assimilation [18], induces oxidative stress by increasing production of reactive oxygen species (ROS) [5,7,19,20], and damages the cells’ cytoskeletons by disrupting organization of microtubules [18]. However, further investigations are needed to clarify the mode of action of BPA on terrestrial plants.

The *Allium* test is a relevant short-term test system for environmental monitoring [21,22]. As a test organism, *A. cepa* has several advantages: its root growth dynamic is very sensitive to pollutants, the mitotic phases of root meristem cells are clearly visible, it has large chromosomes of a stable and reduced number (2*n* = 16), and spontaneous chromosomal damage rarely occurs, allowing easy observation of chromosome aberrations and interpreting the mutagenic potential of pollutant [21,23]. Additionally, *A. cepa* root cells have the metabolic capacity for activating promutagens, thus, in comparison the Ames test addition of S9 mixture (rat liver S9 fraction) is not needed [22]. It is demonstrated that results obtained by *A. cepa* test correlate well with results obtained on other test organisms, including *Daphnia magna* or the V79 cell line [21,23]. Therefore, *A. cepa* is frequently used for ecotoxicity testing and has been adopted by the International Program on Plant Bioassays (IPPB) for monitoring or testing environmental pollutants [23,24].

The aim of this study was to investigate BPA phytotoxicity and the impact of BPA on growth hormone, DNA damage, and oxido-reductive balance in *A. cepa* root cells. By monitoring several biomarkers in root cells in the same experimental set-up we could obtain a better insight into the interplay of assessed parameters and their role in BPA phytotoxicity.

## 2. Results and Discussion

### 2.1. Impact of BPA on A. cepa Root Growth

**Figure 1:**
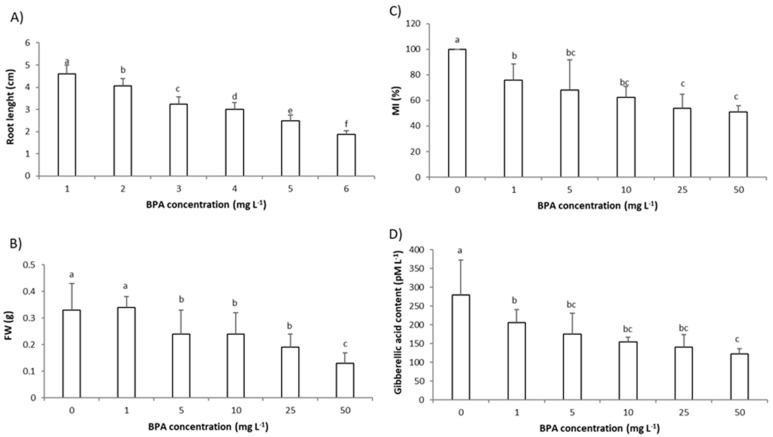
Parameters of root growth and level of plant hormone gibberellic acid (GA_3_) in the root of *Allium cepa* after 3 days exposure to bisphenol A (BPA) in concentration range (1–50 mg L^–1^): (**A**) root length, (**B**) root fresh weight (FW), (**C**) mitotic index (MI) of root meristem cells, and (**D**) the level of GA_3_ in root cells. Data are presented as mean ± SD (n = 5 replicates; MI was calculated on total of 2500 cells per treatment (of 5 replicates) and expressed as %, control is set to 100%). Different letters indicate significant difference (*p* ≤ 0.05). 0—control plants, not exposed to BPA.

As root growth parameters, the root length and root fresh weight (FW) were assessed. Exposure to BPA in a concentration range 1–50 mg L^−1^ for 3 days reduced the growth parameters in a concentration-dependent manner (Figure 1A,B). Even exposure to the lowest applied BPA concentration (1 mg L^−1^) was phytotoxic and significantly reduced root length in comparison to the control plants (Figure 1A). At higher concentrations (25 mg L^−1^ and 50 mg L^−1^), BPA induced changes in the root morphology; roots were curly (bent), brownish, and lost hardness (Figure S1 (Supplementary Materials)).

The negative effect of BPA on root growth was recorded on several plant models. BPA (50 mg L^−1^, 6 days exposure) reduced the root length of *C. arietinum* seedlings [14], the radicle length of *P. sativum* seedlings (2 mg L^−1^, 24 h exposure) [13], and BPA (100 µM (approximately 23 mg L^−1^), 8 days exposure) reduced the root length of *O. sativa* seedlings [5]. A study of *G. max* revealed that BPA (6 mg L^−1^, 7 days exposure) reduced root length at all three growth stages tested, at the seedling stage, flowering and podding stage, and seed-filling stage, and the authors observed that the root growth of the seedling stage was the most sensitive to BPA treatment [12]. Thus, our results are in agreement with previous studies.

The mitotic index (MI) represents the number of dividing cells in a cell cycle. The MI of *A. cepa* roots’ meristem cells is considered a sensitive test for estimating the cytotoxicity of an environmental pollutant [21,22]. In this study, 3 days exposure to the lowest BPA concentration (1 mg L^−1^) resulted in an MI reduction in the roots’ meristem cells (Figure 1C); 3 days exposure to BPA at concentration 1 mg L^−1^ reduced MI to 78% while 3 days exposure to BPA at concentration 50 mg L^−1^ reduced MI to 51% in comparison to control (set at 100%). Since an MI below 20% is considered a lethal effect of the pollutant on the tested plant, and an MI under 50% as sublethal [25], it can be concluded that concentrations of BPA tested in this study were under sublethal concentrations. BPA parallel to a concentration-dependent decrease in MI, decreased % of cells in almost all phase of mitosis (Table 1). Earlier studies also found a decrease in the MI of roots’ meristem cells after exposure to BPA. BPA in concentrations 50, 75, and 100 mg L^−1^ (48 h exposure) reduced the MI of *A. cepa* meristem cells to 64, 38, and 20%, respectively, that is in accordance with our results [17]. A decrease in MI was also observed in root tip cells of *P. sativum* after 3 days exposure to BPA at a concentration 10 mg L^−1^ [13].

The inhibition of root growth can be directly related to plant hormone GA_3_, since 3 days exposure of *A. cepa* roots to the lowest BPA concentration (1 mg L^−1^) also reduced the level of GA_3_ in root cells and the reduction was concentration-dependent (Figure 1D). The relationship between root growth parameters and level of GA_3_ is confirmed by very strong correlation (r); r between root length and GA_3_ level was 0.95, and r between root FW and GA_3_ level was 0.86 (Table S1). Endogenous plant hormones affect plant physiological processes, controlling plant differentiation, development, and growth as well as defense processes by improving plant adaptation to their environment [8,18]. Plant hormone GA_3_ is synthesized and acts in rapid growing tissues such as root tips by promoting cell division in the proliferation zone, thus is closely related to primary roots’ elongation. A reduction in GA_3_ level results in plants with shorter roots and smaller root meristems [26], as is the case in our study.

Previously, it was demonstrated that BPA affected the level of plant hormones in the root of *G. max* at three of the plants’ growth stages tested, the seedling stage, flowering and podding stage, and seed-filling stage [12]. In that study, BPA (6 mg L^−1^, 7 days exposure) reduced the level of plant hormones, GA_3_, indole-3-acetic acid (IAA), and ethylene (ETH) and increased the level of abscisic acid (ABA), and the most prominent effect of BPA on root hormones was observed at seedling stage. In comparison to the mentioned study, in our study BPA reduced the GA_3_ level in plant root at a lower concentration (1 mg L^−1^ vs. 6 mg L^−1^) and shorter exposure (3 days vs. 7 days) confirming the *Allium* test as sensitive model for testing environmental pollutants. More importantly, since in our study the change in GA_3_ level was an early event, the level of GA_3_ can be used as a biomarker of plants’ exposure to BPA.

In response to environmental stimuli hormones regulate plant growth and development [26]. In our study, the correlation between root growth parameters and the level of GA_3_ was very strong (Table S1) indicating that as a response to the unfavorable environmental conditions (presence of BPA), the plant, by reducing GA_3_ levels, inhibits root growth to avoid damage and ensure growth under adverse conditions [5]. There are several possible ways by which BPA could affect GA_3_. BPA could interfere with enzymes involved in GA_3_ biosynthesis or its inactivation, could act directly on the genes, or on GA_3_ receptor level [26]. In addition, Li et al. [12] suggested that an accumulation of ROS induced by BPA could be involved in the change in hormone levels in root cells.

### 2.2. Genotoxic Effect of BPA to A. cepa Root Meristem Cells

The decrease in MI induced by BPA except to hormone GA_3_ can be linked to the direct action of BPA on the DNA molecule that can result in an inhibition of cell progression in the cell cycle until the DNA damage is repaired; cells can be stopped in the G2 phase of cell cycle and prevented from entering mitosis until the DNA damage is repaired [27]. In this study, an increase in cells in interphase already after treatment with the lowest BPA concentration (1 mg L^−1^, 3 days exposure) was observed (Table 1). Thus, we further checked the genotoxic potential of BPA to *A. cepa* root meristem cells. 

**Figure 2:**
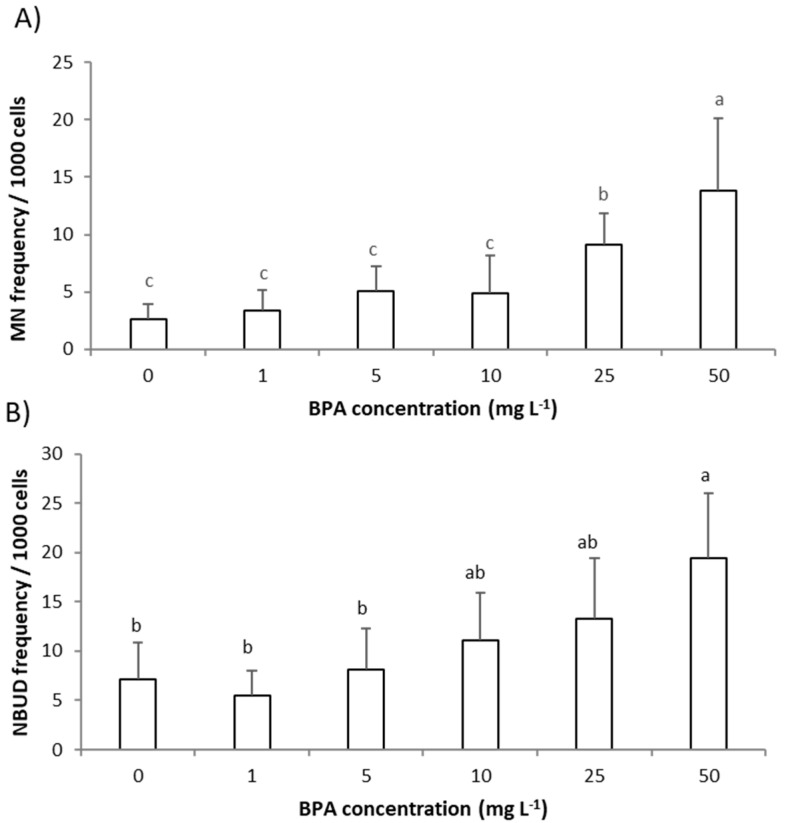
Genotoxic effect of bisphenol A (BPA) to *Allium cepa* roots exposed to BPA (1–50 mg L^–1^) for 3 days. Genotoxicity was assessed as: (**A**) frequency of micronucleus (MNs) and (**B**) frequency of nuclear buds (NBUDs) of interphasic nuclei in meristem cells of *A. cepa* roots. Results are presented as means ± SD (on 4000 cells per treatment). Different letters indicate significant difference (*p* ≤ 0.05). 0—control plants, not exposed to BPA.

The genotoxic effect of BPA to roots’ meristem cells of *A. cepa* exposed to BPA (1–50 mg L^−1^) was estimated by assessing the frequency of MNs and NBUDs of interphasic nuclei in roots’ meristem cells (Figure 2A,B).

Only two higher concentrations of BPA, 25 mg L^−1^ and 50 mg L^−1^, significantly increased frequency of MNs while frequency of NBUDs in meristem cells of *A. cepa* roots was significantly increased only after exposure to the highest concentration of BPA (50 mg L^−1^) (Figure 2A,B). MNs and NBUDs are morphological alterations of interphasic nuclei indicating the mutagenic effects of the tested pollutant [22]. MN is easily observed as a similar structure to the main nucleus but in reduced size, while NBUD is recognized as nuclei carrying nuclear bud. MNs and NBUDs are the result of chromosome aberrations such as chromosome breaks or losses (that cannot be incorporated into the main nucleus during cell cycle), as well as an attempt by the cell to eliminate the excess of DNA that is a result of polyploidization. Previously, Trivedi and Chhaya [17] observed that BPA in concentrations above 50 mg L^−1^ (and 48 h exposure) in *A. cepa* meristem cells induced various chromosomal aberrations (bridge formation, sticky chromosome) as well as binuclear formation. In root tip mitotic cells of *P. sativum* seedlings, BPA (2–25 mg L^−1^, 3 days exposure) produced numerous chromosomal anomalies such as c-mitosis, bridges, laggards, fragments, and sticky chromosomes, and with the increase in BPA concentration the occurrence of chromosomal aberrations increased [9]. Chromosome aberrations (such as laggings, bridges, and sticky chromosomes) can be linked to spindle irregularity caused by BPA treatment. Adamakis et al. [18] on *Triticum turgidum* and *A. cepa* root cells demonstrated that BPA (50 mg L^−1^, 1 h exposure) affected plant mitosis/cytokinesis by disrupting the microtubule organization. Chromosomal aberrations such as fragments and chromosome losses result in micronucleated cells since fragments or entire chromosome cannot be incorporated into the main nucleus during the cell cycle; thus, MNs and NBUDs are an indicator of the direct action of the pollutant on DNA [22]. The results of our study indicate that DNA damage is induced by BPA only at higher concentrations. Detected DNA damage is involved in a reduction in root growth since a strong negative correlation between growth parameters (root length and root FW) and parameters of genotoxicity is observed; r between root length and MNs frequency was −0.92 and r between root FW and MNs frequency was −0.93 (Table S1). Thus, BPA induced root growth inhibition except by GA_3_, at higher BPA concentrations is also mediated through DNA damage. Except for mitotic spindle irregularity, DNA damage and consequent chromosome fragments are linked to the excess production of ROS. Therefore, in the next step we assessed the level of ROS in *A. cepa* roots exposed to BPA.

### 2.3. Impact of BPA on the Level of ROS in A. cepa Root Cells

**Figure 3:**
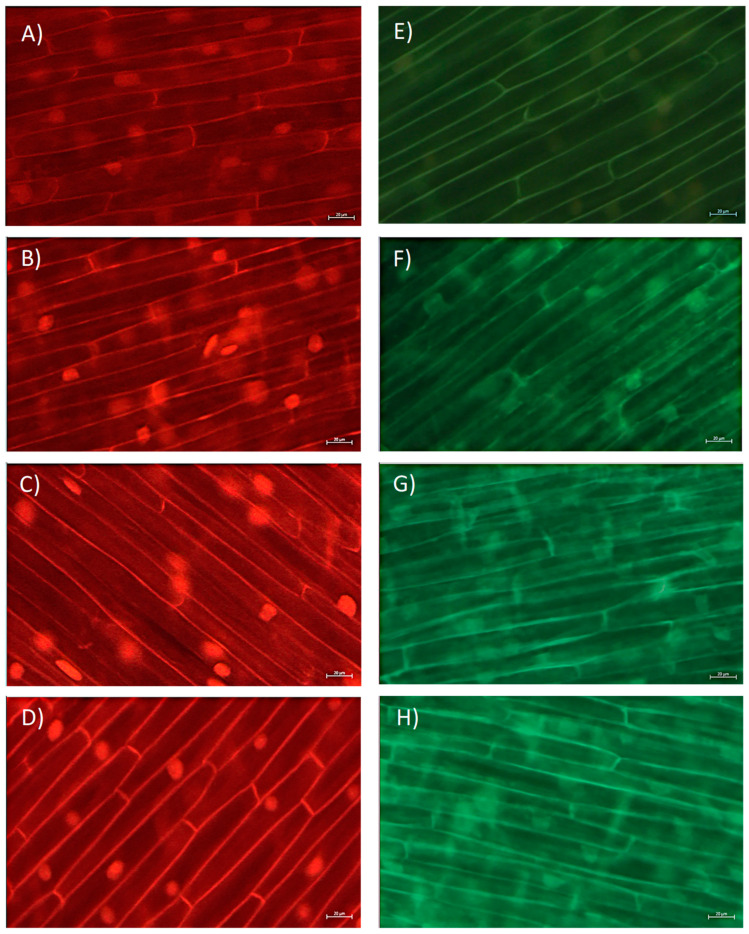
Level of reactive oxygen species (ROS) in situ in root cells of *Allium cepa* exposed to bisphenol A (BPA) in concentration range 1–10 mg L^−1^ for 3 days. Representative fluorescence images of level of: (**A**–**D**) superoxide radical (O_2_^−^) and (**E**–**H**) hydrogen peroxide (H_2_O_2_) in root cells. (**A**,**E**) control; (**B**,**F**) exposure to 1 mg L^−1^; (**C**,**G**) 5 mg L^−1^; and (**D**,**H**) 10 mg L^−1^ of BPA. Scale bar: 20 μm.

**Figure 4:**
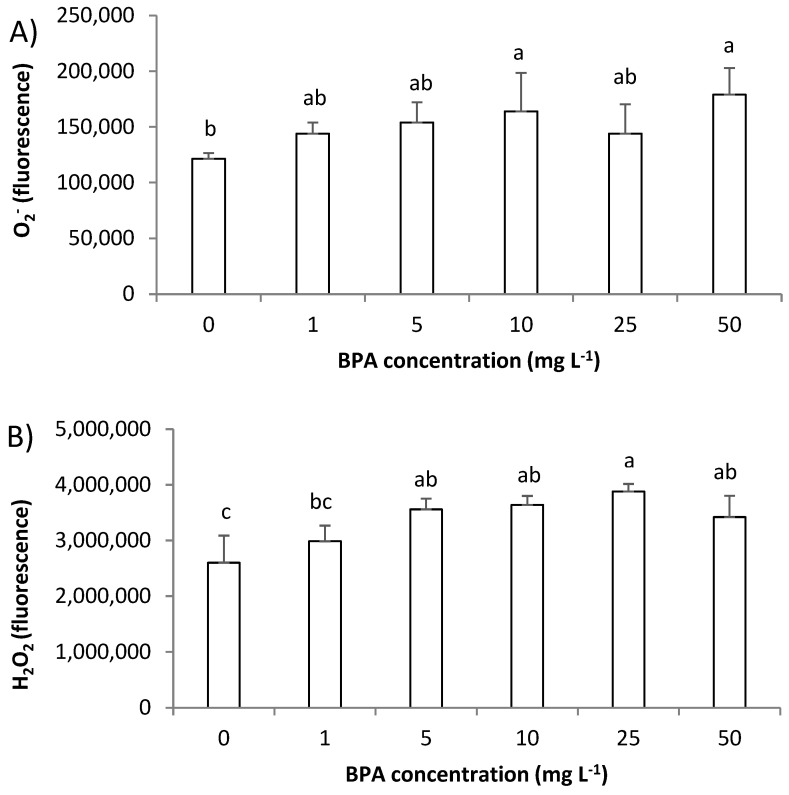
Level of reactive oxygen species (ROS) in root cells of *Allium cepa* exposed to bisphenol A (BPA) in concentration range 1–50 mg L^−1^ for 3 days. Level of: (**A**) superoxide radical (O_2_^−^) and (**B**) hydrogen peroxide (H_2_O_2_). Results are presented as means ± SD (n = 5 replicates). Different letters indicate significant difference (*p* ≤ 0.05). 0—control plants, not exposed to BPA.

In this study, by use of fluorescence microscopy, we followed the level of superoxide radical (O_2_^−^) and hydrogen peroxide (H_2_O_2_). O_2_^−^ was monitored using fluorescent probe dihydroethidium (DHE) that predominately detects O_2_^−^ radicals; meanwhile, for detecting H_2_O_2_, a fluorescent probe 2′,7′-dichlorofluorescin diacetate (H_2_DCF-DA) was used. Although exposure to BPA increased O_2_^−^ level and the increase in red fluorescence was easily observed in the cytoplasm of root cells (Figure 3A–D), a statistically significant increase was observed only after exposure to BPA at 10 mg L^−1^ (Figure 4A). On the other hand, BPA increased the level of H_2_O_2_ already after exposure to 5 mg L^−1^ (Figure 4B), that was observed as increase in green fluorescence in root cells (Figure 3E–H). Thus, BPA induced the formation of ROS, and an increase in H_2_O_2_ was followed by an increase in O_2_^−^ production. Similarly, BPA (6 mg L^−1^, 7-days exposure) induced production of H_2_O_2_ and O_2_^−^ in *G. max* root cells at different plants’ growth stages (seedlings, flowering and podding, and seed-filling stage) [19]. In the study, similar to our observation, at seedling stage BPA induced an increase in H_2_O_2_ at a lower concentration (already at concentration 1.5 mg L^−1^), while an increase in O_2_^−^ at seedling stage was observed at the higher concentration (6 mg L^−1^). An increased H_2_O_2_ production as a response to BPA exposure (0.3 µg L^−1^, 2 days) was observed in seagrass *Cymodocea nodosa* intermediate leaves [5], and exposure to BPA (10 µM, 8 days) increased production of H_2_O_2_ and hydroxyl radicals (OH^−^) in the roots of *O. sativa* seedlings [5]. In our study, a very strong negative correlation of level O_2_^−^ and root length (r was −0.83), and level of ROS and MI (r between O_2_^−^ and MI was −0.82; and r between H_2_O_2_ and MI was −0.89), as well as level of ROS and GA_3_ (r between O_2_^−^ and GA_3_ was −0.86; and r between H_2_O_2_ and GA_3_ was −0.88) (Table S1) indicate that ROS are involved in inhibition of root growth. Thus, the multibiomarker approach used in this study points to the close relationship of GA_3_, MI, and ROS in inhibition of root growth induced by BPA.

### 2.4. Impact of BPA on the Level of Oxidative Damage and Superoxide Dismutase (SOD) Activity in A. cepa Root Cells

Overproduction of ROS leads to the activation of antioxidative defenses and damage of cells’ macromolecules such as membrane proteins and phospholipids which consequently negatively affect plant growth [7,19]. Previous studies on different plant models reported increased oxidative damage of lipids upon exposure to BPA. Additionally, an increase, but also a decrease, in antioxidative defenses is observed. Increased levels of malondialdehyde (MDA), as a marker of lipid peroxidation, and an increase in ascorbate peroxidase but a decrease in SOD activity was observed in seagrass *Cymodocea nodosa* intermediate leaves after 2 days exposure to BPA in concentration of 3 µg L^−1^ [7]. In the study of Ali et al. [5], in roots of *O. sativa* seedlings BPA (10 µM, 8 days exposure) decreased activity of SOD, peroxidase, and catalase and increased activity of ascorbate peroxidase, while MDA was increased at higher BPA concentration (50 µM, 8 days exposure). In *G. max* roots, BPA (6 mg L^−1^, 7 days exposure) increased the activity of the antioxidative enzymes, peroxidase, catalase, and SOD, and increased the level of MDA at plants’ different growth stages (seedlings, flowering and podding, and seed-filling stage) [19].

**Figure 5:**
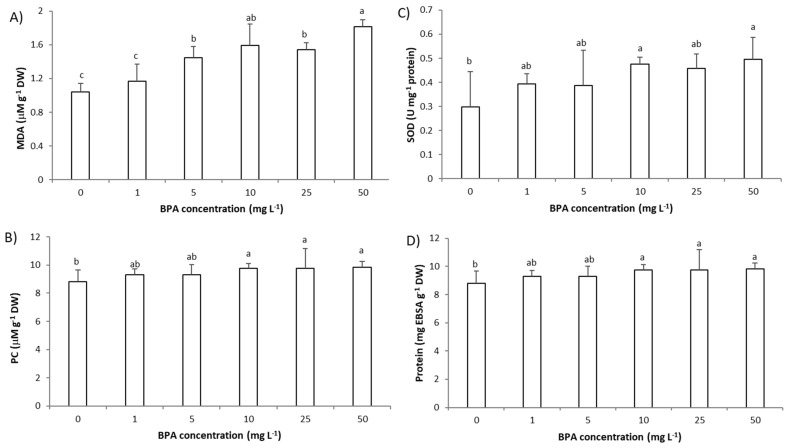
Oxidative stress parameters and level of proteins in *Allium cepa* root cells exposed to BPA (1–50 mg L^−1^) for 3 days. Oxidative stress was monitored as level of: (**A**) malondialdehyde (MDA) as marker of lipid peroxidation, (**B**) protein carbonyls (PC) as marker of oxidative damage to proteins, (**C**) catalytical activity of antioxidative enzyme superoxide dismutase (SOD) and (**D**) total proteins. Results are presented as means ± SD (n = 5 replicates). Different letters indicate significant difference (*p* ≤ 0.05). 0—control plants, not exposed to BPA.

The results of this study indicate that an increased level of ROS, in particular H_2_O_2_ and O_2_^−^, induce damage of cells’ macromolecules, lipids, and proteins. BPA at a concentration of 5 mg L^−1^ (3 days exposure) increased production of MDA (Figure 5A). At a higher concentration (10 mg L^−1^, 3 days exposure), BPA induced the oxidative damage of proteins that was detected as an increase in protein carbonyls (PC) (Figure 5B). The level of O_2_^−^ correlated very strongly with MDA (r was 0.91) and with PC (r was 0.82) and similarly the level of H_2_O_2_ correlated strongly with MDA (r was 0.78) and very strongly with PC (r was 0.83) confirming involvement of ROS in oxidative damage of cells’ lipids and proteins (Table S1). A very strong negative correlation between parameters of oxidative damage (MDA and PC) and growth parameters (root length, root FW and MI; r from −0.82 to −0.97), as well as parameters of oxidative damage (MDA and PC) and GA_3_ (r = −0.94 and −0.96) indicates involvement of oxidative damage of lipids and proteins in inhibition of root growth. A very strong correlation between MDA and parameters of DNA damage (r between MDA and MNs was 0.85 and between MDA and NBUDs r was 0.89) suggests that DNA damage is induced by MDA, probably by formation of MDA-DNA adducts [28,29].

An increase in ROS activates antioxidative defense that can be detected as increased activity of antioxidative enzymes or decrease in their activity if depletion of antioxidative defense occurs [7,19]. Therefore, in this study we assessed the activity of antioxidative enzyme SOD. Exposure to BPA at a concentration 10 mg L^−1^ for 3 days increased SOD activity (Figure 5C). Induction of SOD activity by ROS is confirmed by very strong correlation between SOD and level of O_2_^−^ (r = 0.87). Activation of antioxidative defenses in *A. cepa* root cells can be connected to the increase in protein level (Figure 5D) since protein level correlated well with ROS and oxidative stress parameters (r from 0.88 to 0.97) (Table S1). A strong negative correlation between SOD and growth parameters (root length: −0.91, MI: −0.95 and GA_3_: −0.95) (Table S1) indicate that plant parallel to inhibition of root growth activated synthesis of antioxidative enzymes. This observation is confirmed with a negative, very strong correlation observed between protein level and root growth parameters (root length: −0.95, root FW: −0.93, MI: −0.92 and GA_3_: −0.93). The increase in the protein level could point to activation of synthesis of some other antioxidative enzymes, except SOD.

### 2.5. Impact of BPA on the Level of Phytochemicals in A. cepa Root Cells

Polyphenols are secondary metabolites of plants that are distributed through different plant organs and have multiple functions in plants including plant development, growth, pigment generation, and protection against pathogen attack, as well as defense against stress [30]. In response to abiotic stress, the biosynthesis of polyphenols is usually increased in plants [31,32]. Several studies have demonstrated that polyphenols could attenuate the BPA toxic effect in animal model or animal cells [33]. In BPA-treated rats, *Vincetoxicum arnottianum* extract reduced the toxic effect of BPA that authors attributed to the presence of polyphenols and alkaloids [34]. Polyphenols prevent oxidative stress through several ways; phenolic compounds can directly scavenge ROS or prevent oxidative stress by inhibiting oxidizing enzymes, or by complexing metal ions [30].

To our knowledge, only a few studies have investigated the impact of BPA on the level of polyphenols on plant model [7,16]. In the study of Malea et al., on *Cymodocea nodosa* exposure to low concentrations of BPA (0.3 µg L^−1^ for 1 day) increased the level of total polyphenols (TP), however a higher concentration of BPA (3 µg L^−1^ for 1 day) decreased their level [7], while in roots of *Vicia faba* exposure to a much higher BPA concentration (30 and 120 µg mL^−1^) increased the level of TP [16]. Since plants, in order to cope with stress, synthesize polyphenols, Malea et al. explained the increase in TP with the fact that BPA activated plants’ antioxidant protective mechanism. Since different polyphenols have a different mode of action to reduce stress and protect the plant from stress, in this study we except (beside) following impact of BPA on TP, followed impact of BPA on total flavonoids (TF), total flavonols (TFL), and hydroxycinnamic acids (THA).

**Figure 6:**
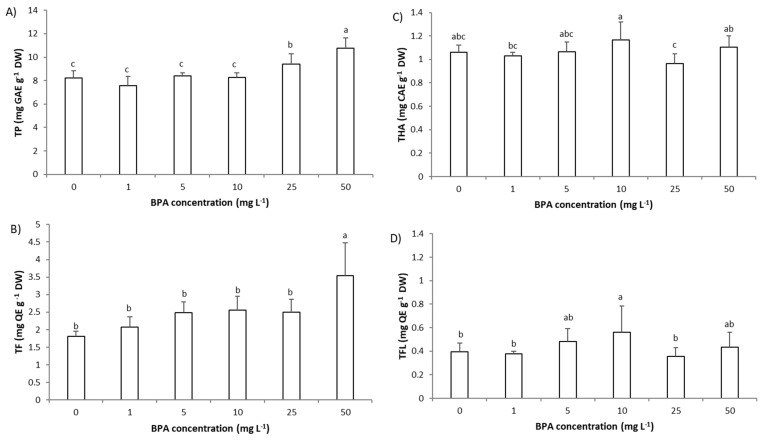
The level of phytochemicals in roots cells of *Allium cepa* exposed to bisphenol A (BPA) (1–50 mg L^−1^) for 3 days. (**A**) level of total polyphenols (TP), (**B**) level of total flavonoids (TF), (**C**) level of hydroxycinnamic acids (THA), and (**D**) level of total flavonols (TFL) Results are presented as means ± SD (n = 5 replicates). Different letters indicate significant difference (*p* ≤ 0.05). 0—control plants, not exposed to BPA.

In root cells of *A. cepa*, BPA (1–50 mg L^−1^, 3 days) affected the level of TP, TF, and TFL, while it had no significant effect on THA (Figure 6A–D). Exposure to BPA resulted in a steady increase in the level of TP and TF, and their level was significantly increased only at higher BPA concentrations, 25 and 50 mg L^−1^, respectively. However, BPA already at a concentration 10 mg L^−1^ increased the level of TFL in root cells of *A. cepa*. These results indicate that in roots, in order to prevent oxidative damage for the plant to survive, synthesis of TP occurred, in particular TF and TFL. This was confirmed with very strong correlations between TF and oxidative stress parameters (r between TF and O_2_^−^ was 0.92, between TF and MDA was 0.94, and between TF and SOD was 0.83). TF also corelated very strongly with TP (r = 0.87) and protein level (r = 0.82). However, TF negatively correlated with root growth parameters (root length: −0.92, root FW: −0.92, MI: −0.82 and GA_3_: −0.84) indicating their close negative relationship. The decrease in GA_3_ level caused by BPA and consequent increase in TF could be linked through common enzymes; gibberellins and flavonoids are characterized by biosynthetic pathways that include similar enzymes [35]. Thus, the plant in a state of stress reassigned enzymes involved in the synthesis of GA_3_ into biosynthesis of flavonoids. In relation to the increased synthesis of TP and TF upon exposure to a high BPA concentration (25 mg L^−1^ and 50 mg L^−1^), it can be concluded that the tested BPA concentrations in this study were not highly toxic to *A. cepa*.

### 2.6. Principle Component Analysis (PCA)

The PCA plots provide an overview of the similarities and differences among different plant treatments as well as the interrelationships between the measured parameters. Factor 1 and Factor 2 described 77.38 and 10.49% of the variance for BPA (0, 1, 5, 10, 25 and 50 mg L^−1^)-treated *A. cepa* root, respectively (Figure S2). Together, the first two Factors represent 87.87% of the total variability. Treatment with two higher concentrations of BPA (25 and 50 mg L^−1^) had strong loadings with frequency of MNs and TP and treatment with the highest concentration of BPA (50 mg L^−1^) had strong loadings with distribution of root meristem cells in interphase, NBUDs, accumulation of O_2_^−^ and H_2_O_2_, MDA, PC, proteins, TF and SOD activity. Treatment with the lowest concentration of BPA (1 mg L^−1^) had strong loadings with roots’ FW, and the control (0 mg L^−1^) had the highest loadings with root length, MI, GA_3_, distribution of root meristem cells in prophase, metaphase, anaphase, and telophase. Moderate BPA treatment (10 mg L^−1^) had strong loadings with THA, TFL, and O_2_^−^. The PCA results confirmed our observation that DNA damage is induced by BPA only at higher concentrations. Additionally, the PCA proved that overproduction of ROS leads to the increase in SOD activity, increase in TP and TF, and induces damage of lipids (MDA) and proteins (PC), as observed in earlier studies [7,16,19]. Parameters of root growth and level of plant hormone GA_3_ as well as the distribution of root meristem cells in prophase, metaphase, anaphase, telophase, and MI were the highest at control samples which indicates the negative influence of BPA on *A. cepa* root cells. The negative effect of BPA on growth, hormone accumulation, MI, and distribution of root meristem cells in prophase, metaphase, anaphase, and telophase were recorded in previous studies on other plant models [5,12,13,14,17].

## 3. Materials and Methods

### 3.1. Chemicals and Preparation of BPA Solution

BPA (4,4′-(propane-2,2-diyl)diphenol; 99% purity), BSA (bovine albumin serum), Coomassie Brilliant Blue G-250, orcein, 2′,7′-dichlorofluorescin diacetate (H_2_DCF-DA), dihydroethidium (DHE), Folin–Ciocalteu reagent, gallic acid (GA), quercetin (Q), caffeic acid (CA), 2,4-dinitrophenylhydrazine and thiobarbituric acid (TBA) and trichloroacetic acid (TCA) were procured from Sigma (St. Louis, MO, USA). Other chemicals used in the study were purchased from Kemika (Zagreb, Croatia) and were of *p.a.* grade or better.

BPA stock solution (200 g L^−1^) was prepared by dissolving BPA in ethanol (96%). Prior to the experiment, BPA test solutions in concentrations: 1, 5, 10, 25, and 50 mg L^−1^ were prepared by diluting BPA stock solution in distilled water (de-water). In BPA test solutions the level of ethanol was less than 0.03%.

### 3.2. Allium Test

The *Allium* test was performed according to Fiskesjo [21,36]. Common onion (*A. cepa* L. var. *argenthea*) bulbs were purchased at a local store. Equal-sized bulbs (1.5–2.0 cm) were selected for the experiment and their outer scales and brownish bottom plate were removed. Root growth was started by placing bulbs into de-water for 48 h. For the treatment experiment, only bulbs with normally developed roots were selected. Bulbs where the roots had visible morphological abnormalities were discharged from the experiment. For each BPA concentration and control (de-water was used as control) 5 bulbs were selected that were transferred to the appropriate BPA test solution or de-water (control). Biological experiment was repeated three times. The experiment was performed at constant room temperature (20 ± 2 °C) and protected from direct sunlight. Due to short exposure time (3 days), there was no need to renew tested solutions in the test tubes.

After 3 days exposure, each bulb was taken from BPA test solution (or de-water), carefully dried with paper towel and morphology of roots were examined (color, consistency and shape/hooks or twists). Then, roots were removed and root length of two the longest of each bulb were measured, and roots FW were assessed. Afterwards, from each bulb three root tips were placed in ethanol: glacial acetic acid (3:1, *v*/*v*) and stored overnight at 4 °C.

For determination of the plant hormone gibberellic acid (GA_3_), oxidative stress parameters, and phytochemicals, the roots were dried in an oven at 60 °C until a constant weight was reached. Roots of each bulb were kept separately. Dried plant material was stored in a dry place at a constant temperature protected from sun.

### 3.3. Determination of Mitotic Index and Micronucleus

Root tips, fixed in ethanol: glacial acetic acid, were first rinsed in de-water and then placed in aceto-orcein (1% orcein in 45% glacial acetic acid) for 15 min at 45 °C [21,23]. Microscopic slides were prepared by squashing stained root tips in aceto-orcein. Per each treatment, five microscopic slides were prepared, and the mitotic index (MI) was calculated on 2500 cells per treatment. Microscope slides were examined under bright-field microscope (CX22LED; Olympus, Tokyo, Japan) at magnification 400× and 1000×. The distribution of cells in each phase of the cell cycle (%) was determined by calculating the total number of cells in interphase, prophase, metaphase, anaphase, and telophase and divided by the total number of counted cells (500 cells per slide/root) and multiplied by 100.

Frequency of micronucleus (MNs) and nuclear buds (NBUDs) was determined on the same microscope slides and nuclear abnormalities were identified as morphological alteration of interphase nuclei [22]. Microscope slides were observed under bright-field microscope (CX22LED; Olympus, Tokyo, Japan) at magnification 400× and 1000×. The analysis of MNi and NBUDs was performed on 4000 cells per treatment point and expressed as frequency on 1000 cells.

### 3.4. Determination of GA_3_ Content

Dried plant material was homogenized in PBS (50 g L^−1^) and centrifuged (10,000× *g* 10 min) and supernatant was collected. In supernatant, level of GA_3_ was determined by use of ELISA commercial kit (MyBioSource, San Diego, CA, USA) according to the producer’s instructions. Absorbance was read on microplate reader (SpectraMax i3x i SpectraMax MiniMax 300; Molecular Devices, San Jose, CA, USA).

### 3.5. Determination of ROS In Situ

The level of ROS in *A. cepa* root cells in situ and by fluorimetry was assessed by employing fluoresce dyes. Level of superoxide radical (O_2_^−^) was assessed using dihydroethidium (DHE) and level of hydrogen peroxide (H_2_O_2_) using 2′,7′-dichlorofluorescin diacetate (H_2_DCF-DA).

Control and BPA-treated roots, fixed in ethanol: glacial acetic acid (3:1, *v*/*v*), were first washed with de-water and then incubated in appropriate fluorescence dye. Fluorescence imaging allows localization of ROS within the cells [37]. For H_2_O_2_ visualization, the root was incubated in 50 μM H_2_DCF-DA for 30 min, and for O_2_^−^ visualization the root was incubated in 10 μM DHE for 30 min. Following incubation in appropriate dye, roots were observed under fluorescent microscope Zeiss Axio Observer 7 equipped with Axiocam 208 camera and Zen 3.4 Pro software (Carl Zeiss, Jena, Germany). To visualize H_2_O_2_, the fluorescent microscope was set at λex 475 nm and λem 535 nm and to visualize O_2_^−^ it was set at λex 555 nm and λem 635 nm. Identical conditions were maintained in all experimental groups. 

To quantify O_2_^−^ and H_2_O_2_ in *A. cepa* root cells, 50 μL of root PBS extract (1 g L^−1^) was mixed with 10 μM of DHE or 50 μM of H_2_DCF-DA, respectively, and fluorescence intensity was read on microplate reader (SpectraMax i3x i SpectraMax MiniMax 300; Molecular Devices, San Jose, CA, USA). To quantify O_2_^−^, the microplate reader was set at λex 535 and λem 635 nm and to quantify H_2_O_2_, the reader was set to λex 485 and λem 535 nm.

### 3.6. Determination of Oxidative Stress Parameters

The level of malondialdehyde (MDA), as a measure of lipid peroxidation, was assessed using the thiobarbituric acid method described by Heath and Packer [38]. The 100 μL of *A. cepa* roots PBS extract (50 g L^−1^) was mixed with 400 μL 0.25% (*w*/*v*) thiobarbituric acid solution containing 10% (*w*/*v*) trichloroacetic acid, heated at 95 °C for 30 min and the reaction was stopped in an ice-bath. The cooled mixtures were centrifuged at 10,000× *g* for 10 min and the MDA content was calculated from the absorbance at 532 nm (correction was performed by subtracting the absorbance at 600 nm for non-specific turbidity) by using the absorption coefficient 155 mM^−1^ cm^−1^. 

The level of protein oxidation was estimated in the reaction of protein carbonyl (PC) groups with 2, 4-dinitrophenylhydrazine as described in Levine et al. [39]. After the 2,4-dinitrophenylhydrazine reaction, the PC content was calculated based on absorbance coefficient of 22 mM^−1^ cm^−1^ and absorbance was measured at 370 nm.

In *A. cepa* root PBS extracts (1 g·L^−1^), SOD activity was determined by use of a commercial kit (Cayman Chemical, Ann Arbor, MI, USA) based on the producer’s instructions. Absorbance was read on microplate reader (SpectraMax i3x i SpectraMax MiniMax 300; Molecular Devices, San Jose, CA, USA).

Total soluble proteins in *A. cepa* roots PBS extract (50 g·L^−1^) were estimated according to Bradford [40]. The absorbance of reaction mixture was read at 595 nm. The protein content was calculated from the calibration curve and expressed as BSA equivalents (BSAE).

### 3.7. Determination of Phytochemicals

Total polyphenols (TP) of *A. cepa* roots PBS extracts were determined with Folin–Ciocalteu reagent according to Zhishen et al. [41]. Volume of 10 μL of root extract was diluted with 790 μL of deionized water and then 50 μL of Folin–Ciocalteu reagent was added. Afterwards, 150 μL Na_2_CO_3_ (1.88 M) was added, and the mixture was incubated for 30 min at 45 °C. The absorbance of the mixture was measured at 765 nm. The TP content was calculated from the calibration curve and expressed as gallic acid equivalent (GAE).

The content of total flavonoids (TF) of root extract prepared in PBS was determined with AlCl_3_ according to the method described by Zhishen et al. [41]. First, a volume of 20 μL *A. cepa* root extract was diluted in 80 μL of dH_2_O and then a volume of 6 μL NaNO_2_ (5%) was added. After 5 min incubation, a volume of 6 μL AlCl_3_ (10%) was added and the mixture was incubated at room temperature for additional 6 min. Afterwards, 40 μL NaOH (1 M) and distilled water were added to a final volume of 200 μL. The absorbance of the reaction mixture was read at 510 nm. The TF content was calculated from the calibration curve and expressed as quercetin equivalents (QE).

The total content of hydroxycinnamic acids (THA) and total flavonols (TFL) of *A. cepa* root extracts prepared in PBS were measured according to the method of Howard et al. [42], using caffeic acid and quercetin as standards. A volume of 25 mL of the *A. cepa* root extract (50 g L^−1^) was mixed with 25 mL HCl (1 g·L^−1^ in ethanol) and 455 mL HCl (2 g L^−1^). The absorbance of the solution was read at 320 and 360 nm, respectively. THA and TFL contents were calculated from the corresponding calibration curves and expressed as caffeic acid (CAE) and quercetin equivalents (QAE), respectively.

All absorbances were read on microplate reader (SpectraMax i3x i SpectraMax MiniMax 300; Molecular Devices, San Jose, CA, USA).

### 3.8. Statistical Analysis

A completely random experimental design was performed. The test of normality and the test of homogeneity of variance was carried out. All results were evaluated using Statistica 14.0.1.25 software package (TIBCO Software Inc., Palo Alto, CA, USA) and were subjected to one-way ANOVA for comparison of means and significant differences were calculated according to Duncan’s multiple range test. The data are presented as the mean ± standard deviations (SD). Pearson’s correlation coefficient and principal component analysis (PCA) between all measured parameters was performed. Data were considered statistically significant at *p* ≤ 0.05.

## 4. Conclusions

The phytotoxicity of BPA to *A. cepa* was evidenced by the decrease in MI of root meristem cells and the consequent decrease in root growth parameters that was recorded already at the lowest applied BPA concentration. The multibiomarker approach used in this study revealed that inhibition of root growth is closely related to decrease in plant hormone GA_3_, since BPA applied at the lowest concentration also decreased the level of GA_3_. Inhibition of root growth and decrease in GA_3_ was followed by production of H_2_O_2_ and O_2_^−^ and activation of antioxidative defenses detected as an increase in SOD activity. Increased production of ROS resulted in oxidative damage to lipids and proteins. Due to increased oxidative stress, plant cells activated the synthesis of polyphenols, TP and TF. In roots’ meristem cells the genotoxic effect of BPA was observed, however at higher BPA concentrations indicating that genotoxic effect of BPA to *A. cepa* roots is probably a consequence of several other biochemical changes including oxidative stress. BPA is phytotoxic to *A. cepa* and its phytotoxicity is mediated through the growth hormone GA_3_ and ROS that consequently lead to oxidative damage of lipids, proteins, and DNA. Due to its genotoxicity, the potential level of BPA in the environment should be monitored.

## Figures and Tables

**Table 1:** Distribution of root meristem cells of *Allium cepa* through phases of cell cycle after 3 days exposure to bisphenol A (BPA) in concentration range (1–50 mg L^−1^).

BPA (mg L^−1^)	Interphase	Prophase	Metaphase	Anaphase	Telophase
0	92.48 ± 1.14 ^cA^	4.32 ± 1.06 ^aB^	2.00 ± 1.06 ^aC^	1.35 ± 0.68 ^aCD^	0.80 ± 0.71 ^aD^
1	94.28 ± 0.94 ^bA^	3.28 ± 0.97 ^bB^	1.27 ± 0.23 ^abC^	0.96 ± 0.74 ^abC^	0.75 ± 0.30 ^aC^
5	94.95 ± 2.04 ^abA^	2.30 ± 0.82 ^bcB^	1.05 ± 0.91 ^abBC^	0.93 ± 0.64 ^abC^	0.60 ± 0.49 ^aC^
10	95.32 ± 0.66 ^abA^	2.76 ± 0.38 ^bcB^	0.85 ± 0.53 ^bC^	0.48 ± 0.23 ^bCD^	0.40 ± 0.24 ^aD^
25	95.96 ± 0.84 ^aA^	2.40 ± 0.40 ^bcB^	0.80 ± 0.49 ^bC^	0.52 ± 0.18 ^bCD^	0.20 ± 0.20 ^aD^
50	96.16 ± 0.36 ^aA^	2.04 ± 0.57 ^cB^	0.75 ± 0.25 ^bC^	0.56 ± 0.43 ^bC^	0.40 ± 0.20 ^aC^

Expressed as % of cells in each phase from the total number of counted cells (n = 500 per replicate). Data are presented as mean value ± SD. Different lower case letters indicate significant difference at *p* ≤ 0.05 within each phase separately. Capital letters indicate significant difference at *p* ≤ 0.05 among phases per treatment (prior to statistical analysis, values were transformed to log).

## Data Availability

Data available on request.

## Supplementary Materials

The following supporting information can be downloaded at: https://www.mdpi.com/article/10.3390/molecules28052046/s1, Figure S1: Morphology of *A. cepa* roots after 3 days exposure to: A- de-water (control), and bisphenol A (BPA) in concentration B- 1 mg L^−1^, C- 5 mg L^−1^, D- 10 mg L^−1^, E- 25 mg L^−1^ and F- 50 mg L^−1^; Figure S2: The principal component analysis (PCA) performed on the correlation matrix of average values of roots length (RL), root fresh weight (FW), mitotic index (MI), level of gibberellic acid (GA_3_), distribution of root meristem cells in interphase, prophase, metaphase, anaphase, and telophase, frequency of micronucleus (MN) and nuclear buds (NBUD), dihydroethidium (DHE) and dichlorofluorescein (DCF) fluorescence (O_2_^−^ radical and H_2_O_2_ detection), levels of malondialdehyde (MDA) and protein carbonyls (PC), superoxide dismutase (SOD) activity, levels of protein in PBS buffer, total polyphenols (TP), total flavonoids (TF), hydroxycinnamic acids (THA) and total flavonols (TFL) for BPA (0, 1, 5, 10, 25 and 50 mg L^−1^) treated *Allium cepa* root samples; Table S1: Pearson’s correlations between measured parameters (roots length (RL), roots’ fresh weight (FW), mitotic index (MI), level of gibberellic acid (GA_3_), distribution of root meristem cells in interphase, prophase, metaphase, anaphase, and telophase, frequency of micronucleus (MN) and nuclear buds (NBUD), O_2_^−^ and H_2_O_2_, levels of malondialdehyde (MDA) and protein carbonyls (PC), superoxide dismutase (SOD) activity, levels of protein in PBS buffer, total polyphenols (TP), total flavonoids (TF), hydroxycinnamic acids (THA) and total flavonols (TFL)).

## Sample Availability

Sample of the tested compound, bisphenol Ais available from the authors.
