# Supplementary material for: Phytotoxicity of Bisphenol A to Allium cepa Root Cells Is Mediated through Growth Hormone Gibberellic Acid and Reactive Oxygen Species
Source: Molecules. 2023 Feb 22;28(5):2046. doi: 10.3390/molecules28052046 (PMC10004651; doi:10.3390/molecules28052046)
Supplement: Supplementary file 1 [file molecules-28-02046-s001.zip › molecules-2216714-supplementary.pdf]

Table S1. Pearson's correlations between mesured parametars (roots length (RL), roots fresh weight (FW), mitotic index (MI), level of gibberellic acid (GA_3), distribution of root meristem cells in interphase, prophase, metaphase, anaphase and telophase, frequency of micronucleus (MN) and nuclear buds (NBUD), level of O_2^- and H_2O_2, level of malondialdehyde (MDA) and protein carbonyls (PC), superoxide dismutase (SOD) activity, level of protein in PBS buffer, total polyphenols (TP), total flavonoids (TF), hydroxycinnamic acids (THA) and total flavonols (TFL)).

	RL	FW	MI	GA_3	Interphase	Prophase	Metaphase	Anaphase	Telophase	MN	NBUD	O_2^-	H_2O_2	MDA	PC	SOD	Protein	TP	TF	THA	TFL
RL	1.00																				
FW	0.97	1.00																			
MI	0.95	0.86	1.00																		
GA_3	0.95	0.86	1.00	1.00																	
Interphase	-0.95	-0.86	-1.00	-1.00	1.00																
Prophase	0.91	0.85	0.95	0.95	-0.95	1.00															
Metaphase	0.90	0.79	0.99	0.99	-0.99	0.94	1.00														
Anaphase	0.87	0.77	0.93	0.94	-0.93	0.80	0.95	1.00													
Telophase	0.87	0.83	0.87	0.85	-0.87	0.74	0.83	0.90	1.00												
MN	-0.92	-0.93	-0.79	-0.78	0.79	-0.75	-0.69	-0.66	-0.70	1.00											
NBUD	-0.91	-0.94	-0.75	-0.75	0.75	-0.67	-0.66	-0.70	-0.73	0.96	1.00										
O_2^-	-0.83	-0.77	-0.82	-0.86	0.82	-0.83	-0.84	-0.77	-0.54	0.72	0.73	1.00									
H_2O_2	-0.80	-0.73	-0.89	-0.88	0.89	-0.86	-0.90	-0.87	-0.90	0.52	0.51	0.59	1.00								
MDA	-0.97	-0.95	-0.92	-0.94	0.92	-0.89	-0.90	-0.88	-0.82	0.85	0.89	0.91	0.78	1.00							
PC	-0.92	-0.82	-0.96	-0.96	0.96	-0.84	-0.96	-0.99	-0.88	0.75	0.77	0.82	0.83	0.91	1.00						
SOD	-0.91	-0.81	-0.95	-0.96	0.95	-0.84	-0.95	-0.97	-0.83	0.76	0.78	0.87	0.79	0.92	0.99	1.00					
Protein	-0.95	-0.93	-0.92	-0.93	0.92	-0.86	-0.90	-0.92	-0.92	0.77	0.83	0.79	0.88	0.97	0.92	0.91	1.00				
TP	-0.84	-0.91	-0.65	-0.64	0.65	-0.62	-0.53	-0.53	-0.63	0.97	0.96	0.61	0.41	0.78	0.62	0.62	0.71	1.00			
TF	-0.92	-0.92	-0.82	-0.84	0.82	-0.83	-0.77	-0.71	-0.62	0.92	0.92	0.92	0.55	0.94	0.79	0.83	0.82	0.87	1.00		
THA	-0.10	-0.11	-0.04	-0.11	0.04	-0.06	-0.12	-0.19	0.07	-0.02	0.18	0.52	-0.03	0.32	0.17	0.25	0.24	0.01	0.32	1.00	
TFL	-0.18	-0.17	-0.20	-0.26	0.20	-0.26	-0.30	-0.32	-0.11	-0.09	0.08	0.52	0.30	0.39	0.26	0.31	0.39	-0.10	0.26	0.88	1.00

Red marked correlations are significant at p≤0.05.

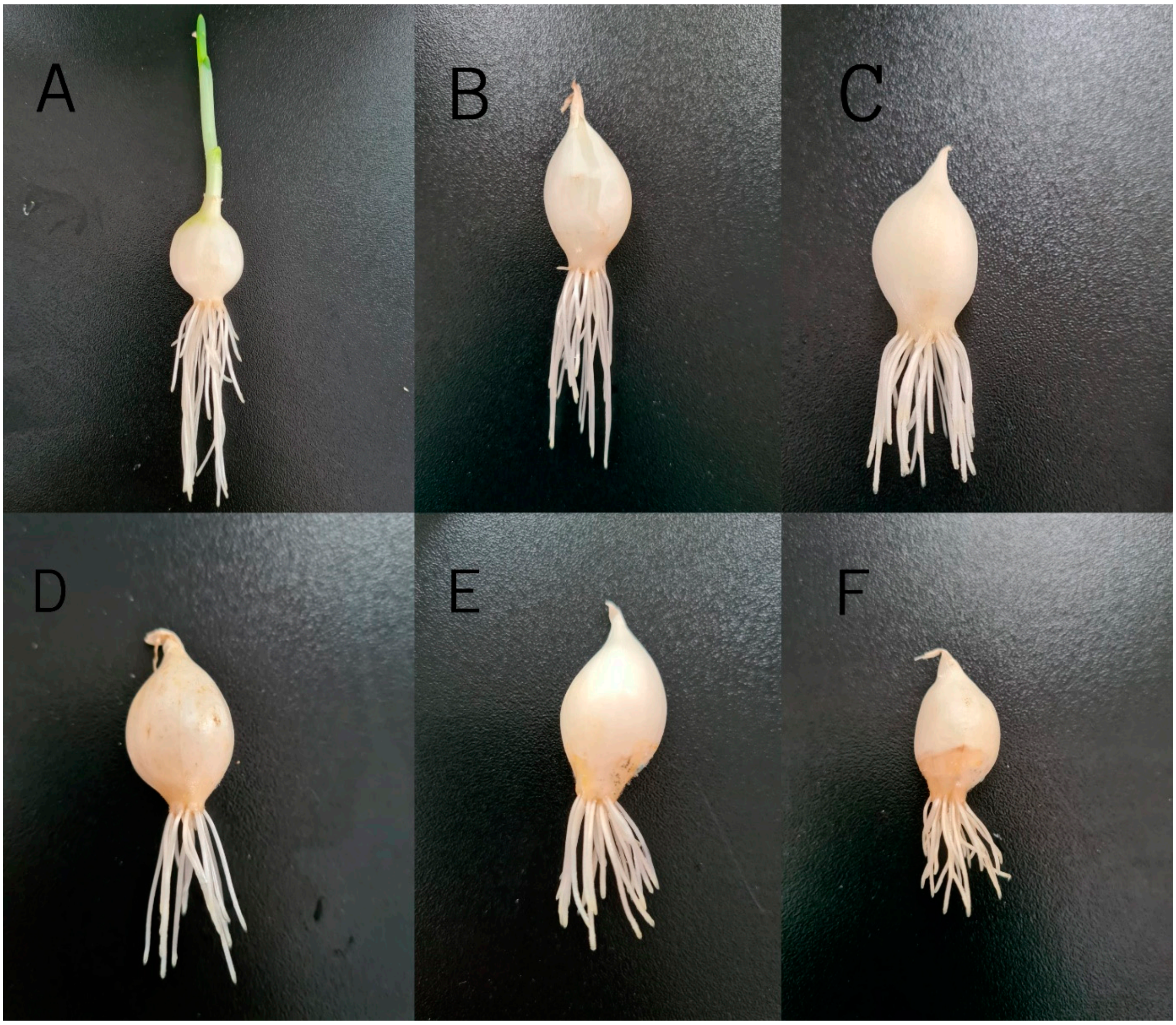

Figure S1. Morphology of *A. cepa* roots after 3-days exposure to: (**A**) de-water (control), and bisphenol A (BPA) in concentration (**B**) 1 mg/L, (**C**) 5 mg/L, (**D**) 10 mg/L, (**E**) 25 mg/L and (**F**) 50 mg/L.

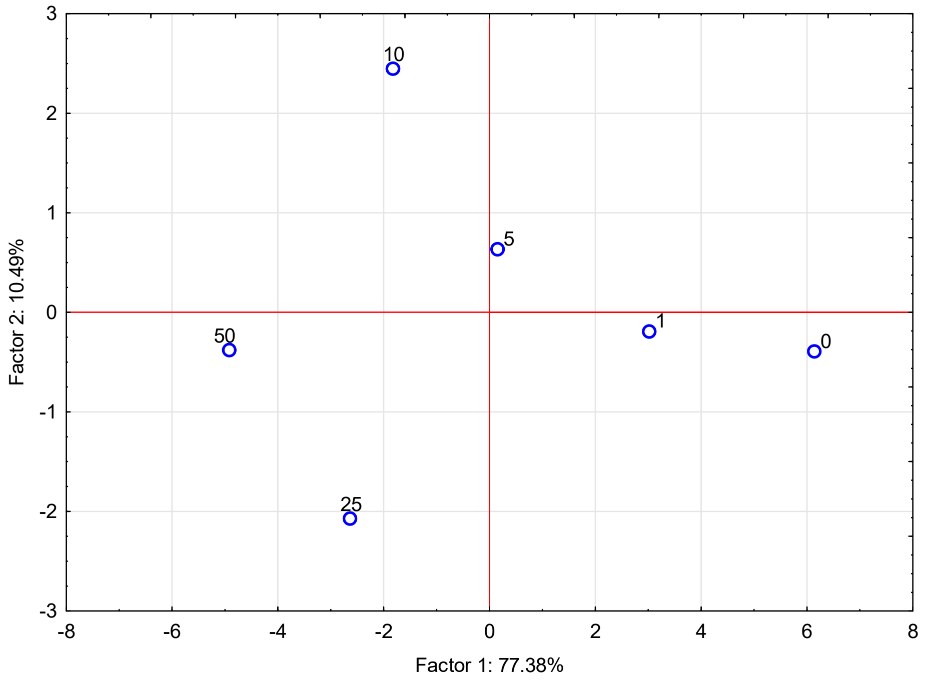

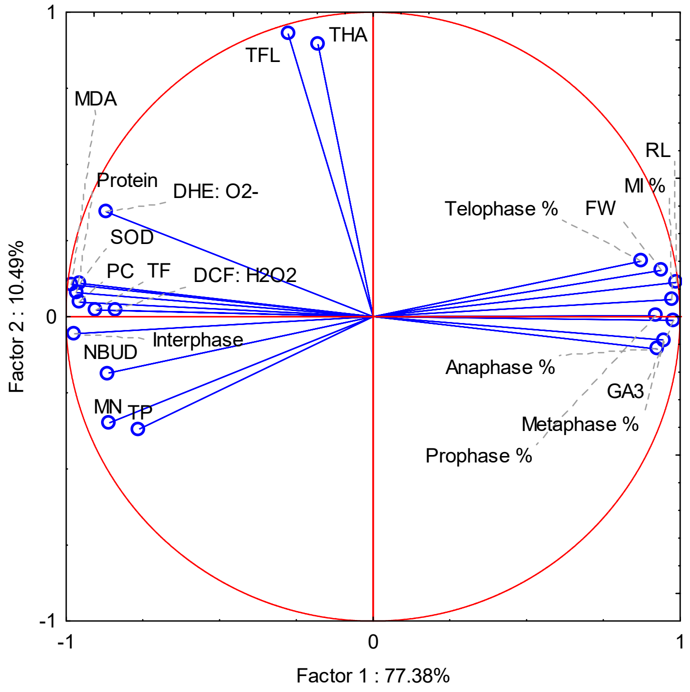

Figure S2. The principal component analysis (PCA) performed on the correlation matrix of average values of roots length (RL), roots fresh weight (FW), mitotic index (MI), level of gibberellic acid (GA_3), distribution of root meristem cells in interphase, prophase, metaphase, anaphase and telophase, frequency of micronucleus (MN) and nuclear buds (NBUD), dihydroethidium (DHE) and dichlorofluorescin (DCF) fluorescence (O_2^- radical and H_2O_2), levels of malondialdehyde (MDA) and protein carbonyls (PC), superoxide dismutase (SOD) activity, levels of protein in PBS buffer, total polyphenols (TP), total flavonoids (TF), hydroxycinnamic acids (THA) and total flavonols (TFL) for BPA (0, 1, 5, 10, 25 and 50 mg/L) treated *Allium cepa* root samples.
